# Antifungal activity of *Burkholderia* sensu lato metabolites against coffee phytopathogens: evidence of chemical diversity

**DOI:** 10.1007/s11274-026-05282-5

**Published:** 2026-09-27

**Authors:** Irma Indira Jiménez-Gómez, Vianey Marín-Cevada, Luis Ernesto Fuentes-Ramírez, Gloria Carrión, Ricardo Enrique Buendia-Corona, Esmeralda García-Díaz

**Affiliations:** 1 Centro de Investigaciones en Ciencias Microbiológicas, Instituto de Ciencias, Benemérita Universidad Autónoma de Puebla, 72592 Puebla, México; 2 Instituto de Ecología, A. C., Carretera antigua Coatepec 351, El Haya, 91073 Xalapa, Veracruz México; 3 Departamento de Fisicomatemáticas, Facultad de Ciencias Químicas Benemérita, Universidad Autónoma de Puebla, Prol. 24 Sur, 72570 Puebla, México; 4 Centro de Química, Instituto de Ciencias, Benemérita Universidad Autónoma de Puebla, Edificio 103H, C.U. Col, San Manuel, Puebla, 72570 México

**Keywords:** Antifungal activity, Secondary metabolites, *Burkholderia* sensu lato, Phytopathogens, Biocontrol

## Abstract

The environmental and health concerns associated with the widespread use of synthetic pesticides have intensified the search for sustainable alternatives to control phytopathogenic fungi. In this context, members of the *Burkholderia* sensu lato group have been recognized for their ability to produce diverse bioactive metabolites with antifungal properties. In this study, the antifungal activity of 36 rhizosphere-derived strains was evaluated against *Hemileia vastatrix*, *Fusarium solani*, *Fusarium oxysporum*, and *Alternaria longissima*. Approximately 80% of the strains inhibited the uredospore germination of *H. vastatrix*, while 37%, 50%, and 45% reduced radial growth of *F. solani*, *F. oxysporum*, and *A. longissima*, respectively. Selected strains (GB99, D335, and D416) produced heat-stable metabolites that retained activity across a wide pH range and showed partial resistance to hydrolytic enzymes. Metabolomic profiling by high-performance liquid chromatography coupled with electrospray ionization quadrupole time-of-flight mass spectrometry (HPLC-ESI-QTOF-MS), performed on metabolites produced by strain GB99, revealed multiple putative antifungal compounds, including burkholdine, occidiofungin and lagriamide related metabolites. Molecular docking, used as an exploratory approach, suggested potential interactions with multiple fungal protein targets. Overall, these findings highlight the chemical diversity and antifungal potential of *Burkholderia* sensu lato metabolites as promising candidates for sustainable biocontrol strategies.

**Supplementary Information:**

The online version contains supplementary material available at https://doi.org/10.1007/s11274-026-05282-5.

## Introduction

Fungi are the primary biological agents responsible for plant diseases. They are responsible for approximately 80% of reported cases, largely due to their diverse infection strategies (Sodikov et al. 2022). Phytopathogenic fungi are commonly classified by their nutritional mode: biotrophs feed on living cells, necrotrophs get nutrients from damaged or dead cells, and hemibiotrophs employ a combination of both strategies (Fei and Liu 2023). Among obligate biotrophs, *Hemileia vastatrix,* the causal agent of coffee leaf rust (CLR), is one of the most destructive pathogens in coffee production. It infects leaf tissues, progressively reducing photosynthetic capacity and yield, a deterioration that worsens over time (Rhiney et al. 2021). Since its first report in Sri Lanka in 1869, CLR has reshaped global coffee cultivation; it was first detected in the Americas in 1970 in Brazil and later caused severe epidemics in Colombia (2008–2013), subsequently spread to Mexico, Peru, and Ecuador, with production losses from 30 to 50% (Avelino et al. 2015; McCook and Vandermeer 2015). More recently, *H. vastatrix* has been detected in regions of North America where it had not previously been reported (Keith et al. 2022). Currently, this pathogen is present in major coffee-producing regions worldwide, generating significant economic losses and increasing production costs (Pandey et al. 2017). In addition to coffee rust fungus, necrotrophic and hemibiotrophic fungi such as *Fusarium* and *Alternaria* species also pose a serious threat to coffee cultivation. In Mexico, *Fusarium solani* and *F. oxysporum* are associated with root rot, wilt, and stem lesions in coffee nurseries and plantations (Gamboa-Becerra et al. 2021), while *Alternaria longissima* causes leaf spots and fruits lesions that reduce both yield and bean quality. The strains of *F. solani*, *F. oxysporum*, and *A. longissima* used in the present study were originally isolated form symptomatic coffee plants in Veracruz, Mexico, confirming their relevance as coffee-associated pathogens. Many *Fusarium* spp. also produce mycotoxins that can pose health risks to humans and animals (Rampersad 2020), and *Alternaria* species are known to generate more than 70 phytotoxins (EFSA Panel on Contaminants in the Food Chain (CONTAM) 2011; Woudenberg et al. 2015) further underscoring the need for their effective control. For decades, the management of these coffee pathogens has relied heavily on chemical fungicides. Copper-based compounds, such as Bordeaux mixture, have been used against CLR since the late nineteenth century, and systemic triazoles (e.g., cyproconazole, tebuconazole) and strobilurins (e.g., azoxystrobin) have been widely applied since the 1970s. Although chemical control remains as an important strategy for managing phytopathogens, the prolonged and intensive use of these compounds has led to the emergence of resistant pathogen strains and raised serious environmental concerns (Pandit et al. 2022). Other management approaches, including resistant crop varieties and cultural practices are also limited by the ability of phytopathogens to rapidly adapt and overcome these strategies (Masreshaw Yirga 2020). Fungal pathogens can rapidly adapt through mechanisms such as target‑site mutations, overexpression of efflux pumps, and the evolution of new virulent races that circumvent host resistance genes (Petit-Houdenot and Fudal 2017; Hawkins and Fraaije 2018). Therefore, there is an urgent need for alternative, more sustainable strategies. Biological control, based on the use of beneficial microorganisms that antagonize pathogens or promote plant defense, has emerged as a promising approach. (Singh et al. 2017). In this context, members of the *Burkholderia* sensu lato group have emerged as promising candidates for biological control of phytopathogens. These bacteria can produce a wide range of bioactive compounds, including enzymes, siderophores, and volatile compounds, which can directly inhibit pathogen growth through antagonistic mechanisms. Their antifungal activity is largely attributed to diverse secondary metabolites encoded by biosynthetic gene clusters (BGCs), encompassing major chemical classes such as non-ribosomal peptides, polyketides, and other structurally diverse compounds. These metabolites have been widely associated with antifungal activity in *Burkholderia* sensu lato strains (Araújo et al. 2017).

The aim of this study was to evaluate the broad‑spectrum antifungal potential of *Burkholderia* strains by deliberately selecting four coffee pathogens with different nutritional strategies: the biotroph *H. vastatrix,* and the necrotrophic/hemibiotrophic fungi *F. solani, F. oxysporum* and *A. longissima*. We assessed the antifungal activity of multiple strains and further investigated the efficacy of secondary metabolites produced by GB99, D335, and D416 under varying conditions of temperature and pH, and in the presence of hydrolytic enzymes. To further characterize the antifungal compounds produced by strain GB99, fermentation extracts were analyzed using HPLC- ESI-QTOF-MS, and the resulting metabolite profile was compared with previously reported antifungal compounds for *Burkholderia* sensu lato. Moreover, an in silico molecular docking approach was performed to explore the capability of the compounds to interact with a set of fungal enzymes and to propose their mechanism of action. Collectively, our findings support the potential of these strains as effective biocontrol agents for sustainable agriculture.

## Materials and methods

### Isolation and identification of *Burkholderia* strains

Rhizosphere soil samples were collected from allegedly healthy plants in multiple locations showing diverse climatic and altitudinal conditions in Puebla State, Mexico. Sampling sites included La Paz Tlaxcolpan, San José del Rincón, San Miguel Atlapulco, Chignahuapan, the Tehuacán-Cuicatlán Biosphere Reserve, Tenampulco, Cuetzalan, Tlatlauquitepec and Citlaltépetl (Pico de Orizaba) The host plant species, coordinates and elevation of each site are provided in Supplementary Table S1. Rhizospheric soil samples were transported to the laboratory in a cooler at 8 °C and processed immediately upon arrival to minimize the time between sampling and processing. Briefly, 1 g of soil was suspended in 9 mL of 10 mM MgSO_4·_7H_2_O and incubated at 30 °C for 30 min under agitation. Serial dilutions (10^–1^ to 10^–4^) were prepared and plated onto semi-selective modified *Pseudomonas cepacia* azelaic acid tryptamine (PCAT) medium. Plates were incubated at 30 °C for 24–48 h, and the morphologically distinct colonies were purified. Preliminary identification was performed by PCR amplification of the 16S rRNA gene fragments using *Burkholderia*-specific primers Bf and Br (Tago et al. 2014, 2015). Genetic diversity and clonal discrimination were evaluated by BOX-PCR using primer BOXA1R (5´-CTACGGCAAGGCGACGCTGACG-3´). Representative isolates were further screened using group-specific PCR assays for plant pathogenic species (*Burkholderia gladioli, B. plantarii,* and *B. glumae*), potential nitrogen-fixing strains, and members of the *Burkholderia cepacia* complex (Perin et al. 2006; Maeda et al. 2006). For taxonomic identification, nearly full-length 16S rRNA genes were amplified using universal primers 27 F (Lane 1991) and 1492R (Weisburg et al. 1991). PCR products (~ 1.4 kb) were purified and sequenced. BLASTn was used for sequence similarity searches. Alignment was performed with MUSCLE in MEGA12 (Kumar et al. 2024).The best fit nucleotide substitution model (TN93 + G + I) was selected by the Bayesian Information Criterion (BIC). A maximum likelihood tree was inferred under this model, with empirical base frequencies, gamma distributed rates, and a proportion of invariant sites estimated from the data. Branch support was evaluated with 1000 bootstrap replicates (Schwarz 1978; Edgar 2004; Camacho et al. 2009).

### Microorganisms and growth conditions

A total of 36 bacterial strains isolated from rhizosphere of wild plants and agriculturally relevant crops from five regions of Puebla State, Mexico, were used in this study. All strains are preserved as glycerol stocks at −80 °C in the culture collection of the Molecular Microbial Ecology Laboratory at ICUAP. Each strain was characterized by growth, purity, and viability in different culture media: Nutrient broth (NB), modified PCAT, and *Burkholderia cepacia* selective agar (BACZ). The modified PCAT medium was prepared based on the formulation described by Burbage et al. (1982) with modifications. The medium contained: 2 g/L azelaic acid, 0.1 g/L MgSO_4_·7H_2_O, 4 g/L KH_2_PO_4_, 4 g/L K_2_HPO_4_, 0.02 g/L yeast extract, 15 g/L agar, pH 5.7. BACZ medium contained (per liter): 2 g/L succinic acid, 0.2 g/L MgSO_4_·7H_2_O, 0.4 g/L KH_2_PO_4_, 0.4 g/L K_2_HPO_4_, 0.02 g/L CaCl_2_, 0.002 g/L Na_2_MoO_4_, 0.01 g/L FeCl_3_, 0.075 g/L bromothymol blue, 15 g/L agar, pH 5.7. All strains were incubated at 30 °C. The phytopathogen *H. vastatrix* was used as the primary target organism. Uredospores were collected from Coffea plants growing in the Abaxomol farm (Coatepec, Veracruz; 1251 m above sea level, 19° 28´ 33.8″ N, 96° 57´ 54.2″ W; Supplementary Fig. S1). The phytopathogenic fungi *Fusarium solani* (strain CBF‑29), *F. oxysporum* (strain CBF‑27), and *Alternaria longissima* (strain CBF‑300) were obtained from the culture collection of the Pilot Plant for the Development of Biological Control Agents, Institute of Ecology, A.C. (INECOL), Xalapa, Mexico. The strains were maintained on potato dextrose agar (PDA) at room temperature (25 ± 2 °C).

### *In vitro* assays of antifungal activity

Bacterial inocula were prepared by recovering the strains from glycerol stock cultures stored at −80 °C. An aliquot of 50 µL from each glycerol stock was transferred to 5 mL of NB or modified PCAT medium and incubated at 30 °C for 48 h under agitation (120 rpm). The resulting bacterial suspension was streaked onto modified PCAT agar plates and incubated at 30 °C for 48 h. A single colony was then transferred to 5 mL of modified PCAT medium and incubated at 30 °C with agitation at 120 rpm. The bacterial suspensions were adjusted to an OD_600_ of 1.56 corresponding approximately to 7.2 × 10^8^–1.25 × 10^9^ CFU/mL. Subsequently, 500 µL of the bacterial suspension was used to inoculate 50 mL of NB or MM9 medium, corresponding to a 1% (v/v) inoculum and an initial bacterial concentration of approximately 7.2 × 10^6^–1.25 × 10^7^ CFU/mL. For the production of secondary metabolites, each of the 36 strains was individually cultured in 50 mL of NB medium supplemented with 10 mL/L of glycerol and 0.05 g/L of succinic acid, for 72 h at 30 °C and 90 rpm. Subsequently, the cultures were centrifuged three times at 5000 rpm for 30 min, and the supernatants were filtered through sterile 0.45 µm syringe filters and stored at 4 °C until use. The bacterial filtrates were used in the germination assays of *H. vastatrix* uredospores. Heat-inactivated fermentation broths were prepared by cultivating strains in 50 mL of modified MM9 medium supplemented with 5 mL/L of glycerol and 0.05 g/L of succinic acid (Caballero-Mellado et al. 2007; Aparicio et al. 2022). The medium composition is provided in the Supplementary Material. Cultures were inoculated with 500 µL of the bacterial suspension described above, corresponding to a 1% (v/v) inoculum and initial bacterial concentration of approximately 7.2 × 10^6^–1.25 × 10^7^ CFU/mL. Cultures were incubated at 30 °C under agitation (90 rpm) for 24, 48, 72 and 84 h. Fermentation broths were then heat-treated in a water bath at 96 °C for 10 min and centrifuged three times at 5000 rpm for 30 min to remove cellular debris. The resulting supernatants were stored at 4 °C until use. The heat-inactivated fermentation supernatants were subsequently used for well-diffusion assays and stability tests.

### Evaluation of supernatant effect on the percentage of *H. vastatrix* uredospore germination

For each treatment, 1 g of *H. vastatrix* uredospores was suspended in 10 mL of bacterial filtrate to obtain a final concentration of approximately 1 × 10^6^ uredospores/mL. Sterile distilled water was used as the reference control, and germination under this condition was established as 100%. The suspensions were incubated in darkness at room temperature (25 ± 2 °C) (Mahfud et al. 2006). The germination percentage of *H. vastatrix* uredospores was evaluated at 24 and 48 h. For each treatment, 20 µL aliquots were analyzed, and 500 uredospores were randomly counted across at least five microscopic fields (400X magnification). All treatments were performed in triplicate. Filtrates that showed inhibitory effects were further evaluated using Coffea leaf discs following the method described by Eskes (1982). Healthy coffee leaves of *Coffea arabica* var. arabica were collected, disinfected, and cut into 1.6 cm discs. Nine 1.6 cm diameter discs obtained from disinfected healthy leaves were placed in airtight containers with a moistened sponge, with the abaxial side facing upwards. Discs were inoculated in the center with a suspension of 1 × 10^6^ uredospores/mL and incubated in the dark at room temperature for 16 h. Subsequently, approximately 120 µL of bacterial filtrate was sprayed onto each leaf disc, whereas the control disc received the same volume of sterile water. Leaf discs were further incubated under the same conditions, and uredospores germination was evaluated after 24  and 48 h of treatment. At each evaluation time, three discs per treatment were randomly selected for microscopic analysis. The discs were air-dried for 35 min, coated a thin layer of transparent nail polish, and dry film was carefully peeled off with tweezers to obtain a leaf print. The leaf prints were mounted on a microscope slide and examined under an optical microscope. For each leaf disc, 165 uredospores were randomly counted across at least five microscopic fields. Uredospore was considered germinated when the germ tube length exceeded the diameter of the uredospore. Germination in the untreated control at each evaluation time was considered 100%, and the percentage of germination for each treatment was calculated relative to the corresponding control (Cabral et al. 2009; Couttolenc-Brenis et al. 2020).

### Dual culture assay

A dual culture assay was conducted in 90 × 15 mm Petri dishes containing PDA medium. A 1 cm × 1 cm mycelial plug from a 7-day-old fungal culture was placed at the center. Five drops (3 µL each) of the standardized bacterial suspension of each strain, prepared as described in the “In vitro assay of antifungal activity” section, were placed equidistantly around the plug (Simonetti et al. 2018). Control plates received five 3 µL drops of sterile NB. The plates were incubated at 25 ± 2 °C in the dark for 11 days. Fungal radial growth was measured daily in millimeters. The antagonistic effect of the bacteria was calculated by determining the percentage of radial inhibition, following the formula: Radial Inhibition (%) = [(Rc—Ri)/Rc] * 100; Rc represents the fungus radius in the control, and Ri represents the radius of fungal growth in the presence of bacteria (Tenorio-Salgado et al. 2013). All experiments were conducted in triplicate.

### Double layer agar method

Twenty microliters of standardized bacterial suspension prepared as described in the “In vitro assays of antifungal activity” were added to the center of a 90 × 15 mm Petri dish containing 8 mL of minimal medium MM9 supplemented with 5 mL/L of glycerol and 0.05 g/L succinic acid. Plates were incubated for 72 h at 30 °C to allow bacterial growth and the production of diffusible secondary metabolites that diffused into the agar matrix. After incubation, the bacterial biomass was carefully removed from agar surface. Five milliliters of chloroform were added to sterile filter paper until complete saturation without overflow, and the impregnated filter paper was placed inside the lid of the Petri dish. The plate containing the agar was then inverted over the lid, to expose the culture to chloroform vapors in a close system for 1 h inside a chemical fume hood. Afterward, a second layer of fungi inoculated modified Luria–Bertani solid medium (yeast extract 5 g/L, casein peptone 10 g/L, agar 15 g/L) was added, previously cooled to approximately 35 °C and inoculated with 50 μL of a 24 h fungal LB culture(liquid) per 20 mL of the medium, was poured over the plates. The plates were then incubated at 25 ± 2 °C until fungal growth developed. During incubation, fungal growth developed uniformly across overlay medium except in areas corresponding to the diffusion of the diffusible secondary metabolites produced during the initial bacterial growth phase. These areas, where fungal growth was absent, were observed as inhibition zones centered on the original bacterial inoculation site. Antifungal activity was evaluated by measuring the diameter of the inhibition zones (mm). All experiments were performed in triplicate (Muñoz-Rojas et al. 2005).

### Well-cut diffusion method

The well-cut diffusion assay was performed using 20 mL of semi-solid LB medium containing 8 g/L agar. After sterilization, the medium was cooled to 35–38 °C and inoculated with 50 µL of a 48-h fungal culture grown in liquid LB medium. The inoculated medium was then poured into 90 × 15 mm Petri dishes and allowed to solidify. Four 5-mm-diameter wells were created at equal distances around the center of each plate using a sterilized cork borer. Each well received 100 μL of heat-inactivated supernatants samples from GB99, D335, and D416, while the control wells received 100 μL of uninoculated liquid medium. Plates were incubated at 25 ± 2 °C. The inhibition halo, defined as the clear zone surrounding each well where no fungal growth was observed, was measured after 48 h for *Fusarium* strains and after 72 h for *A. longissima*. The diameter of each inhibition halo was recorded in millimeters.

### Stability assays

To assess the stability of metabolites produced by *Burkholderia* sensu lato strains, 3 mL of heat-inactivated supernatants were used for each stability treatment. Three independent replicates were used for each treatment. For the thermostability assay, samples were heated to 96 °C or 121 °C for 15 min and then cooled to room temperature. For the pH stability assay, the samples were adjusted to pH 2.0, 4.0, 8.0, or 10.0 using 1 N HCl or 0.5 N NaOH and incubated for 24 h. Subsequently, the pH of each sample was readjusted to 6.0 (corresponding to the initial pH). To evaluate the effect of proteinase K, the samples were treated with proteinase K at a final concentration of 1 mg/mL and incubated at 37 °C for 2 h. The samples were then heated to 96 °C and cooled to room temperature After each treatment, antifungal activity was assessed using the well diffusion method as described previously, with NB medium used as the negative control (Potisap et al. 2018).

### Phytotoxicity assessment on coffee leaves

Healthy coffee leaves were used to evaluate whether the secondary metabolites in the heat-inactivated supernatants of strains GB99, D416, and D335 caused any detrimental effects on *Coffea arabica* leaves. Three leaves were used for each treatment, including the control. Leaf surfaces were disinfected by sequential immersion in 70% ethanol (1 min), 3% sodium hypochlorite (1 min), and 96% ethanol (30 s), followed by three rinses with sterile distilled water. After disinfection, the leaves were placed, abaxial side up, in plastic containers with lids, each lined with a sterile filter paper moistened with 3 mL of sterile distilled water to maintain high humidity. Leaves were treated with heat-inactivated supernatants from strains GB99, D416, and D335, while control leaves received the same volume of sterile distilled water. An initial set of ten spray applications (approximately 12 µL per application) was applied to ensure complete coverage of the leaf surface. Over the following 7 days, the containers were opened daily for 30 min under sterile conditions, and four additional spray applications of the corresponding treatment were applied each day. The leaves were visually assessed for symptoms of phytotoxicity, including chlorosis, necrosis, and tissue damage, and were photographed throughout the experimental period.

### Extraction of metabolites produced by strain GB99 using Amberlite XAD-16 resin

The non-ionic Amberlite XAD-16 resin was chosen for purification because it is suited for capturing non-polar or moderately polar organic molecules from aqueous solutions. We pretreated it by conditioning and adsorption–desorption procedures with modifications (González-Menéndez et al. 2014; Bilal et al. 2018). Briefly, 1.25 g of resin was suspended in 65 mL of sterile distilled water and agitated (150 rpm) for 30 min. The water was decanted, and the resin was washed with 50 mL of methanol, followed by agitation for 60 min under sterile conditions in a laminar-flow hood. Subsequently, the resin was washed three times with sterile distilled water. Five independent 50 mL heat-inactivated fermentation broths from strain GB99 were centrifuged and the supernatants were recovered by decantation and pooled to a final volume of 250 mL. The pretreated resin was added to the pooled supernatant in a 500 mL Erlenmeyer flask and incubated at 28 °C with agitation (180 rpm) for 18 h to promote metabolite adsorption. The resin was recovered by decantation and extracted with 60 mL of methanol under agitation for 3 h. The methanolic extract was recovered by decantation and concentrated under reduced pressure using a rotary evaporator (water bath at 70–75 °C) to approximately 1 mL, then dried at 28 °C until solvent evaporation was complete. The residue was resuspended in 1 mL of an ethanol/water mixture (1:1, v/v) and used in a well-diffusion assay to confirm the presence of metabolites with antifungal activity.

### LC-ESI-QTOF-MS analyses

The obtained extract was analyzed by LC-ESI-QTOF-MS. The compounds in the extract were first separated by liquid chromatography (LC), then ionized softly using electrospray ionization (ESI), and finally detected with a quadrupole time of flight mass spectrometer (QTOF-MS). The hyphenated technique consisted of an Agilent 1260-Infinity HPLC system equipped with autosampler, and temperature controller, coupled to a high-resolution QTOF mass spectrometer and data acquisition software. MassHunter software (v. B.09.00). The equipment uses a binary pump to pressurize solvents A and B and produce the mobile phase mixture with a given exact composition. The samples were analyzed in both positive and negative electrospray ionization mode (ESI ±). Chromatographic separation was achieved on a Synergi Fusion-RP column (80 Å, 4 µm, 150 × 4.6 mm) using the following mobile phase composition gradient: 10% of solvent B (0–5 min), 10–90% of B (5–25 min), 90% B (25–30 min), and 90–10% B (30–35 min), where solvent B was acetonitrile with 0.1% formic acid. Solvent A was deionized water with 0.1% formic acid. Flow rate was 0.7 mL/min and the sample injection volume was 5 µL. Mass spectrometric detection was performed over a mass range of mass to charge ratio (m/z) 50–1700, with the following source parameters: fragmentor voltage 175 V, capillary voltage 3500 V, drying gas temperature 350 °C, N_2_ flow rate 11 L min^-1^, and nebulizer pressure of 60 psi. Similar separation and detection conditions have been used in other investigations (Peris-Díaz et al. 2019; Li et al. 2023). Data were acquired in full-scan mode, generating the so-called total-ion chromatograms. Putative compound identification was performed by a target approach based on comparisons with available databases and literature reporting accurate mass measurements. Data processing and visualization were carried out using MassHunter Qualitative Analysis software.

### Molecular docking

Protein targets were selected from *F*. *solani* and *F. oxysporum* based on the availability of structural information and their relevance in essential cellular processes. Protein structures were retrieved from the UniProt database (http://www.uniprot.org/), prioritizing entries with experimentally determined structures in the Protein Data Bank or high-confidence models from AlphaFold2 (https://alphafold.ebi.ac.uk/) (pLDDT ≥ 80). Active sites were defined using the coordinates of co-crystallized ligands when available or inferred from AlphaFill (https://alphafill.eu/) reference molecules. A total of 13 natural products identified by HPLC-QTOF analysis were selected for molecular docking. Ligand structures were constructed and minimized using Avogadro software (version 1.2.0) (Hanwell et al. 2012) and minimization energy under force field MMFF94s and 10,000 steps followed by further optimization at the semi-empirical PM6 level using Gaussian V.16. The optimized structures were converted to PDBQT format. Docking simulations were performed using AutoDock-FR (Ravindranath et al. 2015) with a genetic algorithm (100 runs; 25,000,000 evaluations per run; grid spacing 0.375 Å). The search space was defined to encompass the predicted binding site of each receptor. For each ligand-receptor pair, the lowest-energy cluster was selected as the representative binding pose. Binding affinity values (ΔG, kcal/mol) were analyzed in R and Python. To assess the distribution and potential selectivity of the identified targets. Protein sequences corresponding to selected receptors were retrieved from the NCBI protein database. Sequences were filtered for fungal taxa and curated by removing redundant entries and those annotated as “putative” or “activator.” Multiple sequence alignments were generated, and maximum likelihood phylogenetic trees were inferred using MEGA12 (Stecher et al. 2026) with 500 bootstrap replicates and nearest-neighbor interchange (NNI) heuristics (Collienne and Gavryushkin 2021).

### Statistical analysis

All experiments were performed in triplicate (biological replicates). Data are represented as mean ± standard error of the mean (SEM). Statistical differences between treatments were analyzed using one-way analysis of variance (ANOVA) followed by Tukey´s honestly significant difference (HSD) post hoc test. For comparisons between two groups, an unpaired t-test with Welch’s correction was used. A significance threshold of *p* < 0.05 was applied for all analyses. Statistical analyses were performed using Graph Pad Prism version 8.

## Results

### Phylogenetic analysis and isolate selection

A maximum likelihood phylogenetic tree (Supplementary Fig. S2) was reconstructed from 1,890 concatenated nucleotide positions of 36 bacterial isolates recovered from the rhizospheres of coffee, pine, bromeliad, canary chili, orchid, fern, agave, blueberry, and corn in Puebla, Mexico. The tree is drawn to scale, with branch lengths representing substitutions per site. The accession numbers of the sequences included in the analysis are listed in Supplementary Table S1. All isolates belonged to *Burkholderia* sensu lato. The majority (23/36) clustered within *Paraburkholderia*, with clear affinities to species such as *P. phytofirmans*, *P. panacisoli*, *P. sediminicola, P. dipogonis, P. terrae, P. aromaticivorans, P. caffeinilytica, P. fynbosensis, P. ginsengisoli, P. nemoris, P. caribensis, P. monticola, and P. hospita.* Ten isolates fell within *Burkholderia *sensu stricto (*B. seminalis, B. gladioli, B. pyrrocinia, B. catarinensis,* and three unidentified *Burkholderia* spp., while three isolates (GB127, GB130, GB137) formed a distinct clade within *Caballeronia* with no close reference species. Based on this diversity, the isolates were further evaluated for their antifungal activity.

### Effect of supernatant on percentage of *H. vastatrix* uredospore germination

Of the 36 bacterial filtrates tested, 27 exhibited a significant reduction on uredospore germination percentage, with a 9–57% germination range. Higher antifungal activity was observed in 10 strains compared to the control (Figure S3 AI, AII), reducing uredospore germination to 45%. Lower germination percentages were observed at 48 h. Strains GA06, GB196, GB99, and D335 consistently showed low germination percentages at both time points. Leaf disc assay confirmed that strains GA06, GB99, D335, and D416 reduced uredospore germination by more than 50% compared with the control. However, no significant differences were detected among the treatments. Since the infection by uredospores occurs within the first 24 h after contact with the leaf surface, subsequent analyses focused on this time. Under these conditions, the filtrate from strain GB99 showed the lowest uredospore germination (21.4%) followed by the treatments with strains GA06 and D416 (Figure S3; BI).

### Dual culture antagonism assay

**Fig. 1:**
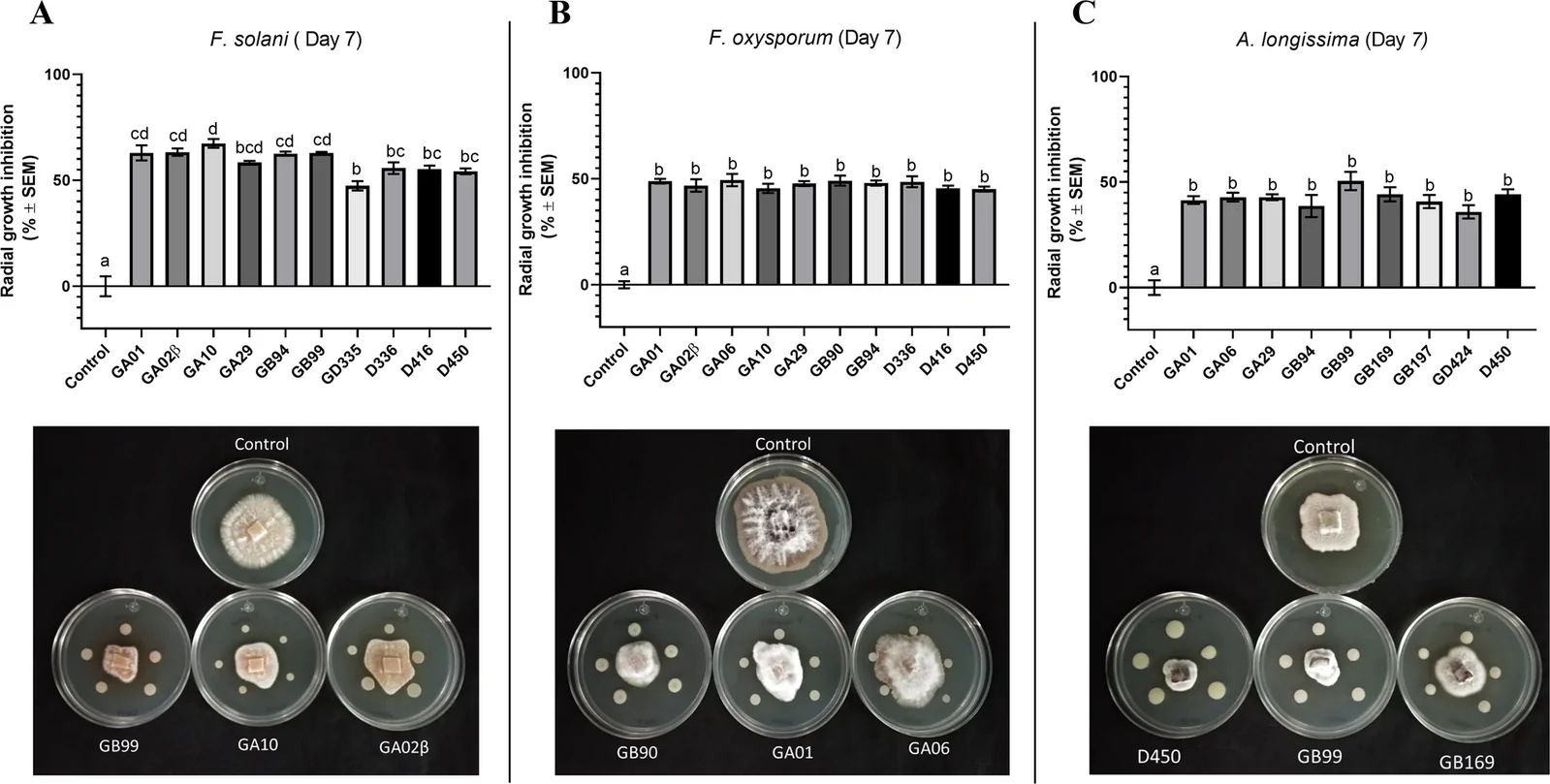
Antagonistic activity against fungi (**A**) *F. solani*, (**B**) *F. oxysporum*, and (**C**) *A. longissimma*, expressed as radial growth inhibition (%) after 7 days of incubation. Only the ten strains that exhibited the greatest antagonistic effect for each fungus are shown. Error bars represent the standard error of the mean ± SEM. Different letters above the bars indicate statistically significant differences among treatments according to the one-way ANOVA followed by Tukey’s multiple comparison test (*p* < 0.05)

Antagonistic activity varied among bacterial isolates depending on the target fungus (Fig. 1). Seventeen strains showed inhibition against *F. solani* from 6% to 67.4%, with strain GA10 exhibiting the highest antagonism, followed by strains GA02β and GA01 (Fig. 1A). For *F. oxysporum*, 23 strains reduced radial growth by 7%−49%, with strains GA06, GB90, and GA01 exhibiting the highest inhibition values (Fig. 1 B). In the case of *A. longissima*, 21 bacterial strains exhibited antagonistic activity, with inhibition ranges of 8%−50.5%. Strains GB99, D450, and GB169 displayed the highest inhibition levels (Fig. 1C).

### Double layer agar assay

Among the five selected strains (GA02β, GA06, GB99, D335, and D416) chosen for their antagonistic properties, distinct inhibition patterns were observed (Table 1). Metabolites produced by strain GB99 generated inhibition zones against all tested phytopathogens, with the strongest effect against *A. longissima* (57 mm), followed by *F. solani* (45 mm) and *F. oxysporum* (30 mm). Strains D335 and D416 also demonstrated antagonistic activity, although in a more selective manner. D335 produced large inhibition zones against *A. longissima* (78 mm) and *F. solani* (75 mm) but showed no inhibition against *F. oxysporum*. Similarly, strain D416 inhibited both *A. longissima* (62 mm) and *F. solani* (54 mm), but exhibited no activity toward *F. oxysporum*. In contrast, strain GA06 showed only a limited inhibition against *F. solani* (14 mm), with no detectable inhibition *of A. longissima* and *F. oxysporum*. Finally, strain GA02β showed no antagonistic activity against any of the tested phytopathogens. This pattern suggests that antifungal metabolite production in some strains may be condition-dependent, potentially requiring specific growth conditions such as solid media. Based on these results, strains GB99, D335 and D416 were selected for subsequent evaluations.

**Table 1:** Inhibition zones produced by *Burkholderia* sensu lato using the double-layer agar technique

Inhibition zones (mm) produced by secondary metabolites of the selected bacteria					
Phytopathogen	GA02β	GA06	GB99	D335	D416
*A. longissima*	**0 ± 0.0**^**a**^	**0 ± 0.0**^**a**^	**57 ± 0.58**^**b**^	**78 ± 0.58**^**c**^	**62 ± 1.53**^**d**^
*F. solani*	**0 ± 0.0**^**a**^	**14 ± 1.15**^**b**^	**45 ± 0.58**^**c**^	**75 ± 0.58**^**d**^	**54 ± 1.15**^**e**^
*F*. *oxysporum*	**0 ± 0.0**^**a**^	**0 ± 0.0**^**a**^	**30 ± 0.58**^**b**^	**0 ± 0.0**^**a**^	**0 ± 0.0**^**a**^

Average inhibition zones diameters produced by *Burkholderia* sensu lato strains against phytopathogenic fungi. Plates were incubated for 48 h for *F. solani* and *F. oxysporum*, and 72 h for *A. longissima*. Data are presented as mean ± SEM. Statistical analysis was performed using one-way ANOVA followed Tukey’s multiple comparisons test (*p* < 0.05). Different letters within the same row indicate significant differences; the letters are assigned independently for each fungus and are not comparable across columns

### Heat inactivated metabolites production

Heat-inactivated supernatants from GB99, D335, and D416 exhibited the antifungal activity against both *F. solani* and *A. longissima*, as evidence by the formation of inhibition zones. Inhibition zones were first detected after 24 h of incubation and reached their maximum diameter after 72 h for strains GB99 and D416, whereas D335 reached its maximum inhibition zone diameter after 84 h (Supplementary Figure S4).

### Stability of antifungal compounds

**Fig. 2:**
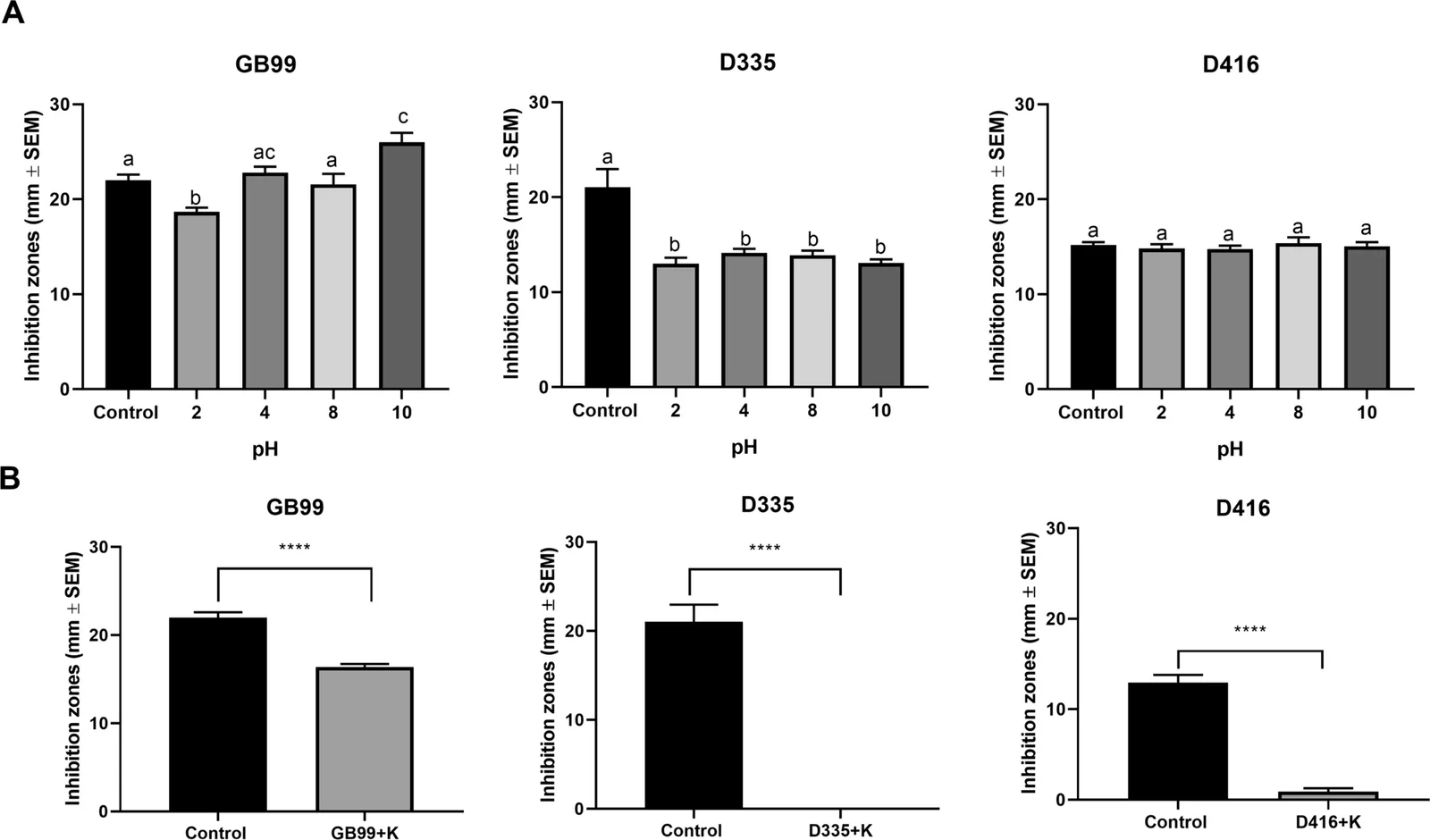
Stability of the antifungal activity of heat-inactivated supernatants from strains GB99, D335, and D416 after pH adjustment and Proteinase K treatment. **A** Antifungal activity after adjusting the pH of the heat- inactivated supernatants to 2, 4, 8 or 10 for 1 h. **B** Antifungal activity after Proteinase K treatment. Error bars represent the mean ± SEM. Different letters above the bars indicate statistically significant differences among treatments according to one-way ANOVA followed by Tukey’s multiple comparison test (*p* < 0.05). Statistical comparisons in panel B were performed using an unpaired t-test with Welch’s correction. **** indicates *p* < 0.0001 compared with the control

Antifungal activity was retained after exposures to temperatures of up to 96 °C but was lost at higher temperatures. After adjustment to pH values ranging from 2 to 10 for 24 h, strain D416 metabolites showed no significant changes compared to the control. GB99 metabolites showed reduced activity when maintained at pH 2 (−15.6%) and increased activity at pH 10. D335 showed a reduction of 30–35% in activity across pH treatments. Proteinase K treatment eliminated antifungal activity in heat-inactivated supernatants from strain D335 and reduced activity in GB99 by 27.5% (Fig. 2B).

### Phytotoxicity assessment

**Fig. 3:**
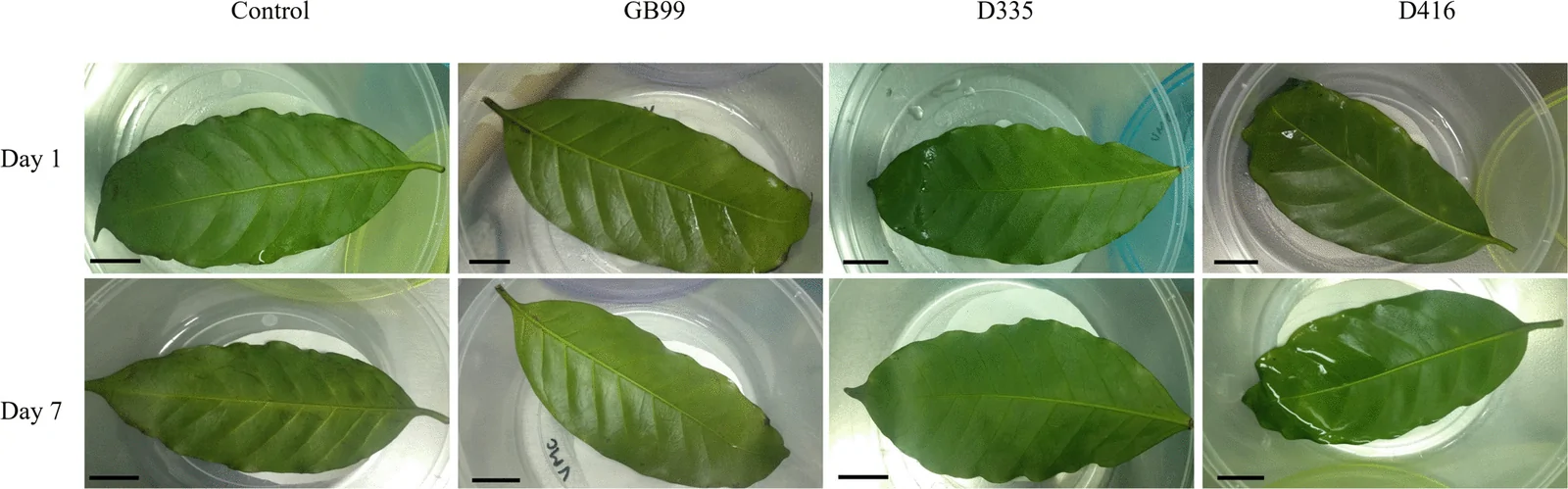
Effect of heat- inactivated bacteria supernatants on coffee leaves. No visible phytotoxic effects were observed after seven days of treatment. Scale bars are shown in each photograph and correspond to 2 cm

No visible phytotoxic effects were observed on leaves treated with heat-inactivated bacterial supernatants from strains GB99, D335, and D416 after seven days. Leaves maintained normal coloration and tissue integrity, with no chlorosis, necrosis, tissue maceration or hypersensitive response were observed (Fig. 3).

### HPLC-ESI-QTOF-MS analysis (ESI—Electrospray Ionization)

**Fig. 4:**
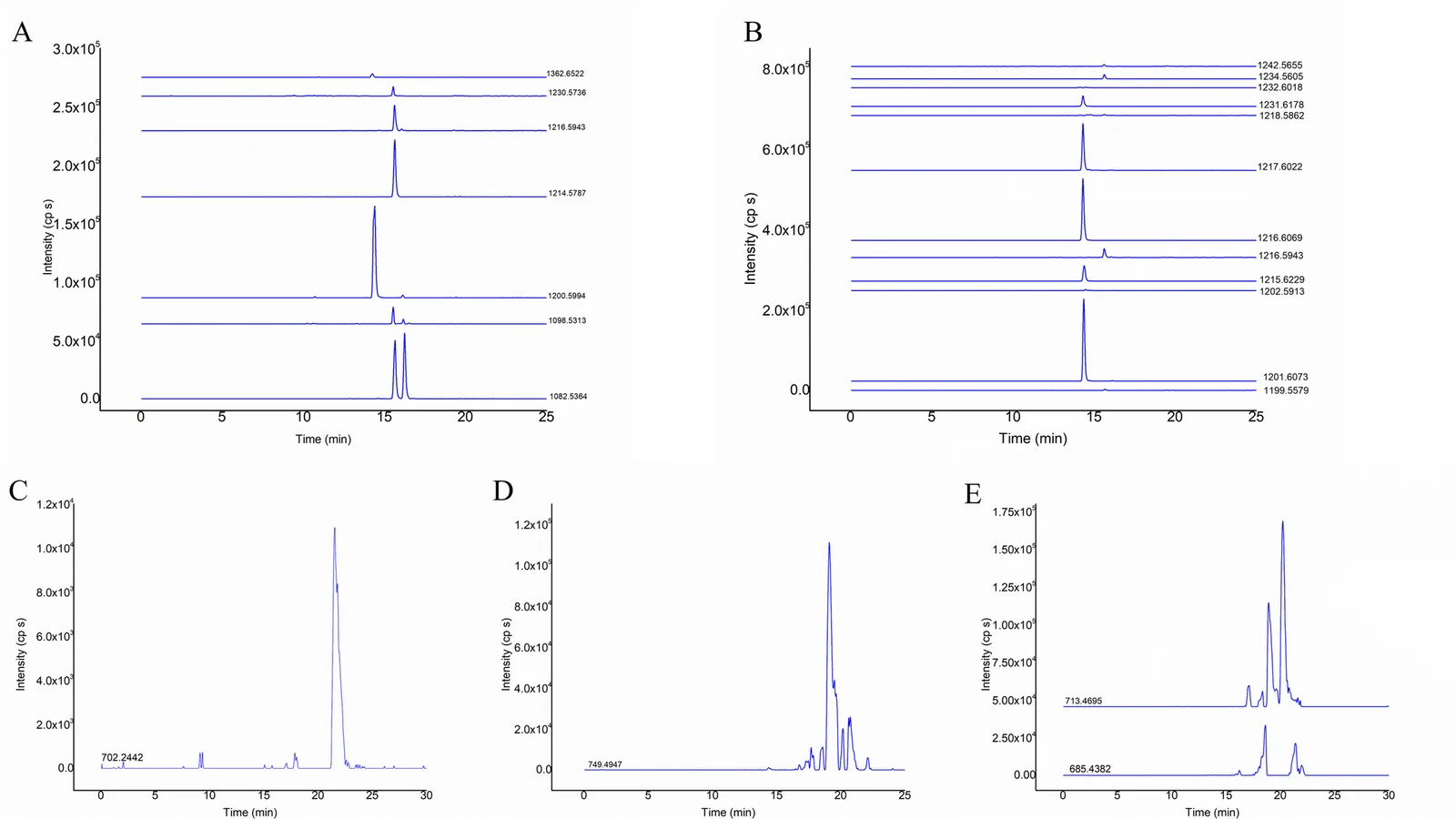
Stacked extracted ion chromatograms (EIC) of selected metabolites detected in positive ionization mode (ESI +). **A** Burkholdine-related ions, **B** occidiofungin-related ions, **C** enacyloxin, **D** lagriamide, and **E** icosalide analogs. The m/z values corresponding to each ion are indicated within each chromatogram. Traces are displayed with a vertical offset for clarification

Liquid chromatography-mass spectrometry analysis allowed for the detection and dereplication of putative metabolites in the extracts, which are shown in Table 2. Dereplication was based on comparison of the observed mass m/z values and molecular formulas with previously reported compounds in the literature. Cyclic polypeptides and polyketides were found to predominate. Extracted ion chromatograms (EIC) (Fig. 4) were obtained with a maximum difference of 10 ppm between the calculated and experimentally observed mass.

**Table 2:** Metabolites detected in extracts from *Burkholderia* strain GB99 by HPLC-ESI-QTOF-MS

Compound family	Molecular formula	Observed m/z (M + H]^+^)	Literature-reported m/z	Reference
Burkholdine type	C_47_H_75_N_11_O_18_	1082.5364	1082.5372	(Tawfik et al. 2010)
	C_47_H_75_N_11_O_19_	1098.5313	1098.5318	(Mullins et al. 2019)
	C_52_H_85_N_11_O_21_	1200.5994	1200.5999	(Mullins et al. 2019)
	C_52_H_83_N_11_O_22_	1214.5787	1214.5792	(Mullins et al. 2019)
	C_52_H_85_N_11_O_22_	1216.5943	1216.5948	(Mullins et al. 2019)
	C_52_H_83_N_11_O_23_	1230.5736	1230.5741	(Mullins et al. 2019)
	C_58_H_95_N_11_O_26_	1362.6522	Not Reported	(Lin et al. 2012)
Enacyloxin	C_33_H_45_NO_11_Cl₂	702.2442	702.2447	(Mullins et al. 2019)
Icosalide	C_34_H_60_N_4_O_10_	685.4382	685.4382	(Jenner et al. 2019)
	C_36_H_64_N_4_O_10_	713.4695	713.4695	(Dangi et al. 2023)
Lagriamide	C_41_H_68_N_2_O_10_	749.4947	749.4947	(Flórez et al. 2018)
Occidiofungin-type	C_54_H_78_N_12_O_19_	1199.5579	Not reported	(Lu et al. 2009)
	C_52_H_86_O_21_N_11_	1201.6073	1200.6027	(Hansanant et al. 2024)
	C_52_H_85_O_22_N_10_	1202.5913	1201.5874	(Hansanant et al. 2024)
	C_53_H_88_O_21_N_11_	1215.6229	Not reported	(Hansanant et al. 2024)
	C_52_N_11_O_22_H_85_	1216.5943	1216.41	(Gu et al. 2011)
	C_53_H_87_O_22_N_10_	1216.6069	Not reported	(Hansanant et al. 2024)
	C_52_H_86_O_22_N_11_	1217.6022	1216.5974	(Hansanant et al. 2024)
	C_52_H_85_O_23_N_10_	1218.5862	1217.5824	(Hansanant et al. 2024)
	C_53_H_88_O_22_N_11_	1231.6178	1230.6134	(Hansanant et al. 2024)
	C_53_H_87_O_23_N_10_	1232.6018	1231.5983	(Hansanant et al. 2024)
	C_52_H_84_N_11_O_21_Cl	1234.5605	Not reported	(Bach et al. 2022)
	C_54_H_84_N_11_O_20_Cl	1242.5655	Not reported	(Bach et al. 2022)

Observed m/z [M + H] ^+^ values were obtained from HPLC-ESI-QTOF-MS data using MassHunter. Literature-reported m/z values correspond to the experimentally observed ions reported by the respective references and are presented for comparison. Literature values are reproduced as reported in the original references and were not recalculated or reinterpreted.

Additionally, 12 occidiofungin-related ions were detected, spanning m/z 1199.5579 to 1242.5655 (Fig. 4B). These ions also exhibited clustering within a defined retention time interval. Other detected metabolites were: enacyloxin (m/z 702.2442), lagriamide (m/z 749.4947), and icosalide analogs (m/z 685.4382 and 713.4695). These compounds showed similar chromatographic behavior, eluting with comparable retention time regions (Fig. 4C-E). Overall, the metabolite profile revealed the presence of multiple structurally related compounds from families summarized in Table 2. Due to the lack of standardized nomenclature for occidiofungin analogs, these metabolites are reported with their minimal formula and exact masses rather than assigned compound names.

### Molecular docking analysis

**Fig. 5:**
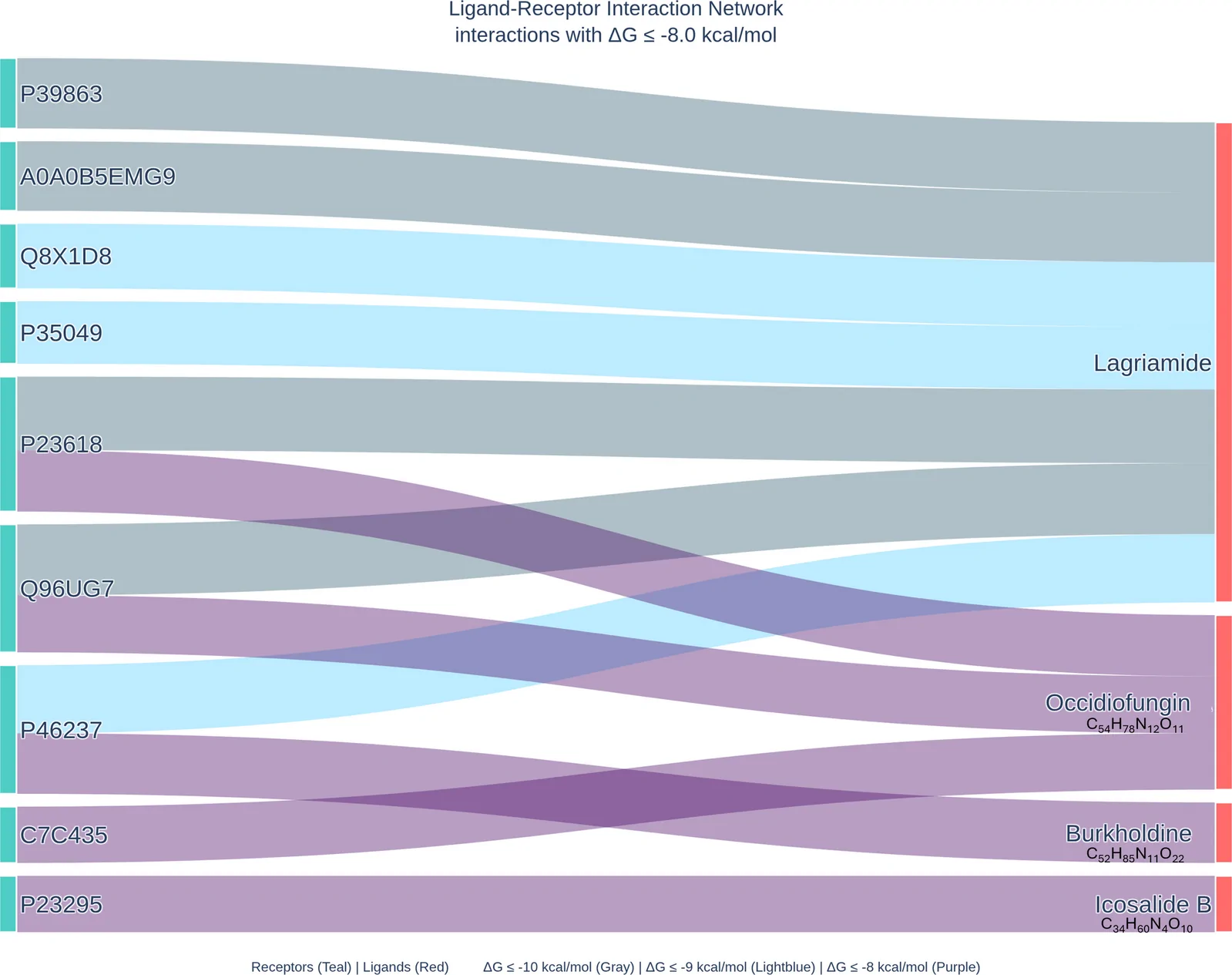
Ligand–Receptor interaction network for the best bioactive compounds (ΔG ≤ − 8 kcal/mol). Receptors are shown in teal columns and ligands in red columns. ΔG ≤ − 10 kcal/mol in gray lines, ΔG ≤ − 9 kcal/mol in light blue lines, and ΔG ≤ − 8 kcal/mol in purple lines

Molecular Docking was performed against 12 protein targets from *F. solani* and *F. oxysporum*, see Table S2, the only bioassay organisms with sufficient structural data available to evaluate potential binding targets for 10 metabolites identified by HPLC-QTOF analysis. *H. vastatrix* lacked reviewed UniProt entries, and *A. longissima* had no database records, precluding structure-based analysis. Binding affinity values (ΔG) ranged from − 10.6 to + 200 kcal/mol (Table S3). Positive ΔG values were considered non-representative and excluded from further analysis. A threshold of ΔG ≤ −8 kcal/mol was used to define strong interactions. Among the evaluated compounds, lagriamide and occidiofungin (C_54_H_78_N_12_O_19_) exhibited the highest number of strong interactions. The interaction network of compounds is shown in Fig. 5. Lagriamide showed strong binding (ΔG ≤ −8 kcal/mol) with seven proteins (Table S3), including a thiamine thiazole synthase (P23618, −10.6 kcal/mol), nitrate reductase (P39863, −10.1 kcal/mol), and the efflux pump FUBT (A0A0B5EMG9, −10.0 kcal/mol). Occidiofungin (C_54_H_78_N_12_O_19_) displayed multitarget binding, interacting with C7C435 (−8.1 kcal/mol), P23618 (−8.8 kcal/mol) and Q96UG7 (−8.2 kcal/mol). Overall, strong interactions were concentrated in a limited number of ligands-receptor pairs, primarily involving lagriamide and occidiofungin.

From the top receptors showed in Fig. 5, phylogenetic analysis for six docking targets retrieved (highly ubiquitous protein P39863, P35049, and Q96UG7 were not considered suitable for selective targeting) 631 fungal homologs distributed across 199 genera, with *Fusarium* (151 spp.) and *Colletotrichum* (139 spp.) as the most represented phytopathogenic genera. Thiamine thiazole synthase (P23618) and the endonuclease III homolog (C7C435) were highly conserved (411 and 336 species, respectively), supporting their suitability as broad-spectrum targets, whereas the FUBT efflux pump (A0A0B5EMG9) and P23295 displayed restricted taxonomic distributions (24 and 13 species), pointing to opportunities for selective inhibition. Nitroalkane oxidase (Q8X1D8) and endonuclease type III (P46237) showed intermediate distributions enriched in *Fusarium* (54 and 20 spp., respectively), consistent with a phytopathogen-biased target landscape (Supplementary File S1).

## Discussion

The results obtained in this study demonstrate that strains belonging to the *Burkholderia* sensu lato group possess significant antifungal potential against multiple phytopathogens, including *H. vastatrix, F. solani*, *F. oxysporum,* and *A. longissima*. The phylogenetic tree confirms a high diversity of *Burkholderia* sensu lato in the rhizospheres of Puebla, with *Paraburkholderia* as the dominant genus (64% of isolates), consistent with its known plant-growth-promoting and rhizosphere-competent lifestyle (Estrada-de los Santos et al. 2018). The clear separation of *Burkholderia* sensu stricto (including *B*. *seminalis*, *B*. *gladioli*, *B. pyrrocinia*) from *Paraburkholderia* supports current taxonomic revisions that restrict the former genus mainly to pathogenic or environmental opportunistic species. The unidentified *Burkholderia* spp. isolates (GA10, GA29, D335, D336, D416), which formed a distinct cluster in the phylogenetic tree, together with the distinct *Caballeronia* clade (GB127, GB130, GB137), likely represent undescribed taxa, warranting whole genome sequencing for formal taxonomic assignment. The absence of host specific clustering (e.g., pine isolates distributed across eight different *Paraburkholderia* species) suggests these bacteria are rhizosphere generalists rather than strict plant specialists.

In agreement with previous reports, members of this group have been widely recognized as environmentally friendly alternatives with both antagonistic and plant growth-promoting capacities (Elshafie et al. 2012; Tenorio-Salgado et al. 2013; Ho et al. 2015; Kunakom and Eustáquio 2019; Depoorter et al. 2021). The inhibition of *H. vastatrix* uredospore germination observed in this study indicates that diffusible metabolites produced by *Burkholderia* sensu lato strains play a relevant role in antifungal activity. However, the magnitude of inhibition was lower than that reported for other bacterial antagonists, such as *Pseudomonas fluorescens* and *Bacillus subtili*s, which achieved inhibition levels above 65% under direct contact conditions (Daivasikamani and Ranajaika 2009). This difference may be explained by the experimental design, particularly the use of cell-free filtrates and simultaneous application with uredospores. Previous studies have demonstrated that the effectiveness of rust control can fluctuate based on the antagonist’s inoculum and the timing of application, reporting higher efficiency when treatments are applied 24 to 72 h prior to pathogen infection (Shiomi et al. 2006; Silva et al. 2012). Several bacterial secondary metabolites, particularly cyclic lipopeptides and polyketides, are known to interfere with fungal development by disrupting membrane, altering cell integrity, and inducing oxidative stress. These compounds can inhibit early stages such as germ tube emergence, suggesting that inhibition is not exclusively dependent on diffusible metabolites. In this context, studies on natural mycoparasites of *H. vastatrix,* such as *Lecanicillium lecanii, Simplicillium lanosaniveum, Akanthomyces muscurius*, have shown that their antagonistic effect relies on the production of hydrolytic enzymes (chitinases, glucanases, and proteases) capable of degrading fungal cell walls and compromising uredospore viability (Jackson et al. 2012; Gómez-de la Cruz et al. 2022). Importantly, these effects are observed only when direct contact between the antagonist and the pathogen occurs. This contrasts with the use of bacterial supernatants in the present study, where such contact-dependent mechanisms are absent.

Comparison of the two experimental systems used in this study further highlights the importance of environmental context when evaluating the antifungal activity of bacterial metabolites. In the uredospore germination assay, uredospores were continuously exposed to bacterial culture filtrates, whereas the coffee leaf disc assay represents a more complex environment in which metabolite activity may be influenced by interactions with the leaf surface, diffusion, degradation, or other physicochemical factors associated with plant tissues. As previously reported, inhibitory effects observed under controlled laboratory conditions may be attenuated in detached leaves or under field conditions, and in some cases, treatment with endophytic bacteria can even increase spore germination or lesion development (Silva et al. 2012). Although the coffee leaf disc assay generally showed lower inhibition than the uredospore germination assay, comparison of the corresponding treatments between the two experimental systems revealed no statistically significant differences. Therefore, the observed trend should be interpreted as reflecting environmental complexity’s influence on metabolite performance rather than as evidence of reduced efficacy under leaf-disc conditions. Importantly, uredospore germination remained below 50% across all treatments, indicating that bacterial metabolites consistently restricted germination under the evaluated conditions. These observations suggest that the antagonistic effect is not attributable to a single metabolite, but rather to a combination of factors, including active metabolism, continuous production of antifungal compounds, and possible cell-associated or volatile-mediated interactions. Similar observations have been reported previously (James et al. 2016) underscoring that the overall antifungal effect of beneficial bacteria often results from the combined action of multiple complementary mechanisms rather than a single bioactive compound.

From an applied perspective, these findings suggest that different biological control strategies may be more suitable for different target pathosystems and environmental conditions. Cell‑free metabolite formulations offer practical advantages, including ease of storage, stability during formulation, and the absence of viable microorganisms, making them particularly attractive for foliar applications. In contrast, living *Burkholderia* sensu lato strains may provide additional benefits through continuous metabolite production, rhizosphere colonization, competition for ecological niches, and the induction of plant defense responses. Rather than representing alternative strategies, metabolite-based products and living bacterial inoculants could be considered complementary components of integrated disease management programs. For CLR, preventive application of antifungal metabolites before uredospore germination may represent an effective strategy to interfere with one of the earliest and most vulnerable stages of pathogen establishment. Nevertheless, greenhouse and field studies will be required to determine the optimal formulation, application timing, persistence under environmental conditions, and compatibility with existing disease management practices. Overall, metabolites produced by *Burkholderia* sensu lato strains show considerable promise for managing CLR, although their practical performance will ultimately depend on formulation, application strategy, and environmental conditions.

The results of the dual-culture assays reveal that several strains exhibited a broad-spectrum antagonistic activity. However, a differential response among phytopathogens was observed, with *F. solani* showing greater susceptibility than *F*. *oxysporum*. This variability may be associated with differences in fungal physiology, cell wall composition, or metabolic adaptability. Notably, members of the *Burkholderia* sensu lato group have been increasingly recognized as an ecological alternative for plant disease management (Kunakom and Eustáquio 2019; Depoorter et al. 2021). Beyond their antagonistic activity against phytopathogens, these bacteria are also known for their roles as plant-growing promoting rhizobacteria, contributing to plant development and health (Elshafie et al. 2012; Tenorio-Salgado et al. 2013; Ho et al. 2015). For instance, *Burkholderia* sp. strain HQB-1 has been reported to inhibit pathogens such as *F*. *oxysporum, Colletotrichum gloeosporioides, Botrytis cinerea,* and *Curvularia fallax*, while simultaneously promoting plant growth through the production of phenazine-1-carboxylic acid (Xu et al. 2020). Similarly, *Burkholderia contaminans* AY001 demonstrated antifungal activity against *F. oxysporum* and *F. solani,* as well as plant-growth promoting traits in tomato, including the induction of plant defense response (Heo et al. 2022). To our knowledge, this study represents the first report of secondary metabolites from *Burkholderia* sensu lato evaluated against *H. vastatrix* as well as the first evaluation of these bacteria against *A*. *longissima*. These findings highlight the potential of the studied strains as biocontrol agents against economically important phytopathogens and support their application within environmentally sustainable agricultural strategies.

Metabolites produced by *Burkholderia* strains include hydrolytic enzymes (e.g., chitinases, lipases, and β−1,3-glucanases) as well as secondary metabolites such as phenazine, which can disrupt fungal cell wall integrity and lead to irreversible damage (Elshafie et al. 2012; Xu et al. 2020; Ahmad et al. 2022; Heo et al. 2022). The combined action of these compounds likely contributes to the observed antifungal effects. In previous studies, antifungal activity of bacterial metabolites has been evaluated using a broad range of volumes from 5–10 µL to milliliter scale additions (up to 2 mL), at different concentrations, directly to the plates where phytopathogens are cultured. In our case, inhibition of phytopathogens was achieved using 100 µL of supernatant within this experimental range, which was sufficient to produce clear inhibition zones. Similar observations were reported by Chávez-Ramírez et al. (2020), who used a 5 µL drop of supernatant that produced an inhibition halo approximately equivalent to the size of the applied drop. In contrast, in the present study, the application of 100 µL generated inhibition zones that extended well beyond the initial area occupied by the drop, suggesting that the active metabolites are diffusible in the medium. This behavior can also indicate the possible contribution of proteinaceous compounds, implying that one or more compounds may be responsible for the antifungal activity.

Stability assays indicate that antifungal activity is associated with chemically diverse compounds. Retention of activity under heat and across a broad pH range suggests the presence of non-proteinaceous metabolites such as lipopeptides and polyketide-derived compounds. Compounds such as bacteriocins, lipopeptides, and secondary metabolites (e.g., CF66I) are known for their thermal stability and wide pH tolerance (Quan et al. 2006; Li et al. 2007; Bharti et al. 2012; Xue et al. 2022). However, the partial loss of activity after the proteinase K treatment indicates that proteinaceous compounds also contribute to antifungal activity. Similar behavior has been reported in *B. cenocepacia*, which reduces antifungal efficiency through enzymatic degradation (Rojas-Rojas et al. 2018). In contrast, certain enzymes, such as lipases, may lose efficacy at temperatures above 50 °C (Jegannathan et al. 2009), supporting the coexistence of multiple classes of bioactive compounds with different stability profiles.

Importantly, the absence of phytotoxic effects on coffee leaves supports the compatibility of these metabolites with plant tissues, and their potential applicability in agriculture.

The metabolomic profile revealed a chemically diverse secondary metabolism dominated by cyclic lipopeptides and polyketide-derived compounds. In particular, the detection of multiple ions associated with burkholdine and occidiofungin variants supports the co-production of structurally related analogs, consistent with the biosynthetic flexibility of hybrid PKS-NRPS pathways (Lu et al. 2009; Lin et al. 2012; Chen et al. 2013; Wang et al. 2016; Wu et al. 2023). These compounds have been reported to exhibit antifungal activity through mechanisms such as membrane disruption and interference with essential cellular processes. The detection of additional metabolite features consistent with enacyloxin, lagriamide and icosalide analogs, further highlights the metabolic versatility of the strain. Overall, these findings support a model in which antifungal activity is not attributable to a single compound but rather to the synergistic or additive effect of multiple chemically diverse metabolites.

Molecular docking analysis provided insights into the potential mechanisms underlying the antifungal activity of the putative assigned metabolites. Lagriamide and occidiofungin showed the strongest and most frequent interactions with multiple fungal protein targets, supporting a multitarget mode of action. Lagriamide exhibited high binding affinity toward thiamine thiazole synthase (P23618), suggesting interference with thiamine pyrophosphate (TPP) biosynthesis and central metabolism (Ruiz-Roldán et al. 2008). Strong interactions were also observed with nitrate reductase (P39863), indicating potential effects on nitrogen assimilation, and with the efflux-related protein FUBT (A0A0B5EMG9), suggesting impaired detoxification and transport processes (Bell et al. 2015). Additional interactions with endonuclease type III (P46237) and nitroalkane oxidase (Q8X1D8) further support the disruption of stress response and oxidative metabolism (Fitzpatrick 2017; Rungrattanakasin et al. 2018). Overall, these results indicate that antifungal activity may arise from the simultaneous disruption of multiple metabolic pathways. Occidiofungin displayed a comparable interaction profile, supporting a complementary mechanism of action. The multitarget nature of these interactions may reduce the likelihood of resistance development.

Phylogenetic analysis of the protein targets revealed differences in conservation and distribution. Conserved proteins such as P23618 suggest potential for broad-spectrum activity, whereas more restricted targets, including FUBT, indicate opportunities for selective inhibition. Other target proteins, such as P46237 and Q8X1D8, showed intermediate distribution enriched in phytopathogenic fungi. These findings highlight a balance between broad-spectrum activity and target specificity and support lagriamide as a relevant antifungal scaffold, particularly for *Fusarium*-targeted strategies. However, further experimental validations are required to confirm the biological relevance of these interactions.

Taken together, these results provide an integrated view of the antifungal potential of *Burkholderia* sensu lato strains, combining biological, chemical, and in silico evidence. Bioassays, stability analyses, and metabolomic profiling indicate that antifungal activity is associated with a chemically diverse set of metabolites, including both proteinaceous and non-proteinaceous compounds. Molecular docking suggests a potential multitarget mode of action, in which structurally diverse metabolites may interact with key fungal pathways related to energy metabolism, nutrient assimilation, stress response, and detoxification. These computational predictions support the notion that antifungal activity arises from the combined effects of multiple compounds rather than a single metabolite; however, their biological relevance must be confirmed through future in vitro enzyme inhibition assays or in vivo experiments. From an applied perspective, the physicochemical stability of these metabolites and the absence of phytotoxic effects support their potential for biocontrol applications. However, differences between in vitro and plant-based assays highlight the influence of environmental conditions and application strategy.

Notably, the strain GB99 consistently exhibited the most favorable profile across all assays: broad-spectrum antifungal activity, strong inhibition in both direct and diffusible metabolite-based tests, production of chemically diverse metabolites as shown by HPLC-QTOF analysis, and partial tolerance to heat and pH variations. Its absence of phytotoxic effects on coffee leaves further supports its premise as a safe and effective biocontrol candidate. These combined attributes position GB99 as a particularly promising strain for future development. Further evaluation under greenhouse and field conditions is required to confirm their effectiveness and optimize their use in sustainable agriculture.

## Supplementary Information

Below is the link to the electronic supplementary material.

Supplementary file1 (DOCX 16 KB)

Supplementary file2 (XLSX 40 KB)

Supplementary file3 (PNG 2777 KB)

Supplementary file4 (DOCX 4479 KB)

Supplementary file5 (DOCX 182 KB)

Supplementary file6 (DOCX 142 KB)

Supplementary file7 (DOCX 27 KB)

Supplementary file8 (DOCX 3001 KB)

Supplementary file9 (DOCX 18 KB)

## Data Availability

No datasets were generated or analysed during the current study.
