# Supplementary material for: Antifungal activity of Burkholderia sensu lato metabolites against coffee phytopathogens: evidence of chemical diversity
Source: World J Microbiol Biotechnol. 2026 Sep 27;42(10):556. doi: 10.1007/s11274-026-05282-5 (PMC13616859; doi:10.1007/s11274-026-05282-5)
Supplement: Supplementary file 1 [file 11274_2026_5282_MOESM1_ESM.docx]

| **Composition of modified MM9 culture medium** | |
| --- | --- |
| Component | Final concentration or amount (per 1 L of culture medium) |
| Ferric citrate (6g/L) | 1 mL |
| Goodies solution | 2.5 mL |
| Succinic acid**^a^** | 0.05 g |
| 10 X M9 solution | 100 mL |
| MgSO_4_·7H_2_O (1M) | 1 mL |
| Glycerol**^a^** | 5 mL |
| Agar**^b^** | 15 g |

| Composition of Goodies solution | |
| --- | --- |
| Compound | Final concentration or amount (per 1 L of culture medium) |
| ZnCl_2_ | 50 mg |
| MnCl_2_·4H_2_O | 30 mg |
| BO_3_H_3_ | 300 mg |
| CoCl_2_ | 200 mg |
| CuCl_2_·2H_2_O | 10 mg |
| NiCl_2_·6H_2_O | 20 mg |
| NaMoO_4_·2H_2_O | 30 mg |

| 10 X M9 solution | |
| --- | --- |
| Compound | Final concentration or amount (per 1 L of culture medium) |
| Na_2_HPO_4_ | 70 g |
| KH_2_PO_4_ | 30 g |
| NaCl | 5 g |
| NH_4_Cl | 10 g |

**^a^** Sterilized separately and added after autoclaving.

**^b^** Added only for preparation of solid medium.
