# Supplementary material for: Antifungal activity of Burkholderia sensu lato metabolites against coffee phytopathogens: evidence of chemical diversity
Source: World J Microbiol Biotechnol. 2026 Sep 27;42(10):556. doi: 10.1007/s11274-026-05282-5 (PMC13616859; doi:10.1007/s11274-026-05282-5)
Supplement: Supplementary file 2 [file 11274_2026_5282_MOESM4_ESM.docx]

**Supplementary Figure S2.** Maximum‑likelihood phylogenetic tree of 36 bacterial isolates from rhizosphere samples in Puebla, Mexico, and reference strains of *Burkholderia sensu lato*. The tree was inferred from 1,890 concatenated nucleotide positions (1st, 2nd, 3rd, and non‑coding sites) using the Tamura‑Nei model with a discrete Gamma distribution (5 categories, shape parameter = 0.1325). Bootstrap support values (1,000 replicates) are shown at nodes; branches with <50% support are collapsed. The initial tree was obtained by selecting the topology with the superior log‑likelihood between a Neighbor‑Joining tree (based on Tamura‑Nei distances) and a Maximum Parsimony tree (shortest among 10 random‑starting searches). Evolutionary analyses were conducted in MEGA12 (Kumar et al., 2024).
