# Supplementary material for: Antifungal activity of Burkholderia sensu lato metabolites against coffee phytopathogens: evidence of chemical diversity
Source: World J Microbiol Biotechnol. 2026 Sep 27;42(10):556. doi: 10.1007/s11274-026-05282-5 (PMC13616859; doi:10.1007/s11274-026-05282-5)
Supplement: Supplementary file 3 [file 11274_2026_5282_MOESM5_ESM.docx]

 Figure S3. Effect of bacterial metabolites on *H. vastatrix* uredospores germination. AI and AII, effect of direct contact between *H. vastatrix* uredospores and bacterial filtrates after 24 h and 48 h of exposure, respectively. Only the 10 strains with the greatest antagonistic effect are shown. BI and BII, effect of bacterially sprayed filtrates on coffee leaf discs with the inoculation of *H. vastatrix* uredospores after 24 h and 48 h of incubation, respectively. Germination percentages are presented as mean ± SEM, followed by Tukey’s HSD multiple comparison test (*p*<0.05). Different letters above the bars indicate statistically significant differences among treatments.
