# Supplementary material for: Antifungal activity of Burkholderia sensu lato metabolites against coffee phytopathogens: evidence of chemical diversity
Source: World J Microbiol Biotechnol. 2026 Sep 27;42(10):556. doi: 10.1007/s11274-026-05282-5 (PMC13616859; doi:10.1007/s11274-026-05282-5)
Supplement: Supplementary file 4 [file 11274_2026_5282_MOESM6_ESM.docx]

 **Supplementary Figure S4**. Antifungal activity of heat-inactivated supernatants from strains GB99, D335, and D416 against *F. solani* and *A. longissima* at different incubation periods. Data are presented as mean ± SEM. For each fungal species, different lower letters indicate significant differences across incubation periods within the same bacterial strain, according to the one-way ANOVA followed by Tukey’s HSD multiple comparison test (*p*<0.05).
