# Supplementary material for: Antifungal activity of Burkholderia sensu lato metabolites against coffee phytopathogens: evidence of chemical diversity
Source: World J Microbiol Biotechnol. 2026 Sep 27;42(10):556. doi: 10.1007/s11274-026-05282-5 (PMC13616859; doi:10.1007/s11274-026-05282-5)
Supplement: Supplementary file 5 [file 11274_2026_5282_MOESM7_ESM.docx]

| Strain ID | GenBank  Accession  number | Plant species (host) | Sampling site  (locality, Puebla) | Latitude (N) | Longitude (W) | Altitude  (m a.s.l.) |
| --- | --- | --- | --- | --- | --- | --- |
| GA01 | PZ447919 | *Zea mays* | La Paz  Tlaxcolpan | 18°54′17.57″ | 98°12′35.31″ | 2061 |
| GA02B | PZ447921 | *Fabaceae* | La Paz  Tlaxcolpan | 18°54′37.35″ | 98°12′01.29″ | 2093 |
| GA06 | PZ447920 | *Zea mays* | La Paz  Tlaxcolpan | 18°54′34.79″ | 98°12′00.38″ | 2120 |
| GA10 | PZ447923 | *Zea mays* | La Paz  Tlaxcolpan | 18°54′58.14″ | 98°12′58.14″ | 2125 |
| GA29 | PZ447925 | *Zea mays* | La Paz  Tlaxcolpan | 18°54′34.39″ | 98°12′52.36″ | 2061 |
| GA51B | PZ447924 | *Filicopsida* | Chignahuapan | 19°50′01.76″ | 97°59′33.71″ | 2257 |
| GB23 | PZ447922 | *Filicopsida* | Chignahuapan | 19°50′01.55″ | 97°59′34.07″ | 2260 |
| GB42 | PZ447926 | *Filicopsida* | Chignahuapan | 19°50′01.21″ | 97°59′34.21″ | 2258 |
| GB45 | PZ447927 | *Filicopsida* | Chignahuapan | 19°50′02.24″ | 97°59′34.36″ | 2256 |
| GB53 | PZ447928 | *Filicopsida* | Chignahuapan | 19°50′02.04″ | 97°59′34.19″ | 2257 |
| GB62 | PZ447929 | *Filicopsida* | Chignahuapan | 19°50′02.09″ | 97°59′34.24″ | 2257 |
| GB70 | PZ447930 | *Pinus* sp*.* | Chignahuapan | 19°50′01.88″ | 97°59′34.24″ | 2258 |
| GB90 | PZ447931 | *Pinus* sp*.* | Chignahuapan | 19°50′01.75″ | 97°59′33.99″ | 2258 |
| GB91 | PZ447932 | *Pinus* sp*.* | Chignahuapan | 19°50′02.23″ | 97°59′33.85″ | 2254 |
| GB81 | PZ447933 | *Pinus* sp*.* | Chignahuapan | 19°50′01.35″ | 97°59′34.86″ | 2263 |
| GB87 | PZ447934 | *Pinus* sp*.* | Chignahuapan | 19°50′02.82″ | 97°59′35.45″ | 2258 |
| GB99 | PZ447936 | *Agave* sp. | Chignahuapan | 19°50′02.94″ | 97°59′35.87″ | 2260 |
| GB100 | PZ447935 | *Agave* sp | Chignahuapan | 19°50′03.38″ | 97°59′36.40″ | 2261 |
| GB127 | PZ447937 | *Orchidaceae* | Chignahuapan | 19°50′03.79″ | 97°59′36.70″ | 2262 |
| GB130 | PZ447938 | *Orchidaceae* | Chignahuapan | 19°50′04.69″ | 97°59′37.09″ | 2260 |
| GB137 | PZ447939 | *Bromelia*  sp. | Chignahuapan | 19°50′05.21″ | 97°59′37.39″ | 2261 |
| GB148 | PZ447940 | *Bromelia*  sp. | Chignahuapan | 19°50′05.48″ | 97°59′37.75″ | 2262 |
| GB152 | PZ447941 | *Bromelia*  sp. | Chignahuapan | 19°50′06.00″ | 97°59′38.30″ | 2265 |
| GB169 | PZ447942 | *Pinus* sp*.* | Chignahuapan | 19°50′06.74″ | 97°59′38.91″ | 2268 |
| GB183 | PZ447943 | *Pinus s*p. | Chignahuapan | 19°50′07.10″ | 97°59′39.25″ | 2270 |
| GB190 | PZ447944 | *Pinus s*p. | Chignahuapan | 19°50′07.46″ | 97°59′39.45″ | 2271 |
| GB197 | PZ447945 | *Pinus s*p. | Chignahuapan | 19°50′07.84″ | 97°59′39.62″ | 2272 |
| GB203 | PZ447946 | *Pinus s*p. | Chignahuapan | 19°50′01.27″ | 97°59′58.50″ | 2262 |
| D328 | PZ447947 | *Vaccinium*  *corymbosum* | Cuetzalan | 20°00′42.72″ | 97°31′18.97″ | 957 |
| D335 | PZ447948 | *Capsicum*  *pubescens* | Cuetzalan | 20°00′45.05″ | 97°31′19.62″ | 967 |
| D336 | PZ447949 | *Capsicum*  *pubescens* | Cuetzalan | 20°00′45.64″ | 97°31′19.13″ | 975 |
| D409 | PZ447950 | *Coffea* sp. | Cuetzalan | 20°00′45.49″ | 97°31′16.06″ | 995 |
| D416 | PZ447951 | *Allium sativum* | Cuetzalan | 20°00′47.79″ | 97°31′21.75″ | 980 |
| D422 | PZ447952 | *Orchidaceae* | Cuetzalan | 20°00′32.07″ | 97°31′07.79″ | 986 |
| D424 | PZ447953 | *Orchidaceae* | Cuetzalan | 20°00′32.09″ | 97°31′07.89″ | 985 |
| D425 | PZ447954 | *Zantedeschia* sp*.* | Cuetzalan | 20°00′32.11″ | 97°31′09.28″ | 975 |

**Supplementary Table S1.** Geographic location, altitude, and host plant species of the rhizosphere sampling sites in Puebla, Mexico.
