# Supplementary material for: Antifungal activity of Burkholderia sensu lato metabolites against coffee phytopathogens: evidence of chemical diversity
Source: World J Microbiol Biotechnol. 2026 Sep 27;42(10):556. doi: 10.1007/s11274-026-05282-5 (PMC13616859; doi:10.1007/s11274-026-05282-5)
Supplement: Supplementary file 6 [file 11274_2026_5282_MOESM8_ESM.docx]

Table S2. Putative molecular targets from *F. solani* and *F. oxysporum* selected for docking analysis.

| UniProt ID | Protein Name | Biological Function | Structure Source | PDB ID | Quality (pLDDT) | Binding Site | |
| --- | --- | --- | --- | --- | --- | --- | --- |
| *Fusarium solani (n = 4 proteins)* | | | | | | |  |
| C7C435 | 2-Methylcitrate synthase, mitochondrial | Catalyzes synthesis of (2S,3S)-2-methylcitrate from propionyl-CoA and oxaloacetate. Detoxifies propionyl-CoA. | AlphaFold | NA | 92.94% | Residues: 351, 349, 403 (AlphaFill) | |
| P78717 | Ubiquitin-conjugating enzyme E2 2 | E2 ubiquitin-conjugating enzyme. Catalyzes monoubiquitination of histone H2B for epigenetic regulation. | AlphaFold | NA | 96.44% | Autosite | |
| Q96UG7 | Cyanide hydratase | Catalyzes hydration of cyanide to formamide. Important for infection of cyanogenic plants. | AlphaFold | NA | 92.88% | Residues: 57, 54, 164, 133, 190 (AlphaFill) | |
| Q9C1K0 | Pea pathogenicity protein 2 | Pathogenicity factor contributing to ability to cause disease on pea plants. | AlphaFold | NA | 96.31% | Residues: 153, 40, 194, 65, 132 (AlphaFill) | |
| *Fusarium oxysporum (n = 8 proteins)* | | | | | | |  |
| Q8X1D8 | Nitroalkane oxidase | Catalyzes oxidative denitrification of neutral nitroalkanes. Protects against toxic compounds. | PDB | 2C0U | Experimental | Co-crystallized ligand | |
| P23295 | NADP nitrous oxide-forming nitric oxide reductase | Nitric oxide reductase. Reduces nitrate/nitrite to N2O. Detoxification mechanism. | PDB | 7DVO | Experimental | Co-crystallized ligand | |
| P35049 | Trypsin | Serine protease. Protein degradation and processing. | PDB | 2VU8 | Experimental | Co-crystallized ligand | |
| P39863 | Nitrate reductase [NADPH] | Key enzyme in nitrate assimilation. Reduces nitrate to nitrite using NADPH. | AlphaFold | NA | 86.62% | Residues: 287, 233, 178, 131, 127, 177 (AlphaFill) | |
| P46237 | Endoglucanase type C | Endohydrolysis of β-D-glucosidic linkages. Plant cell wall degradation. | PDB | 4OVW | Experimental | Co-crystallized ligand | |
| P9WER6 | Fusoxypene synthase | Bifunctional sesterterpene synthase. Produces fusoxypenes A, B, C and α-astellatene. | AlphaFold | NA | 84.19% | Residues: 450, 454, 556, 552, 465, 612, 523 (AlphaFill) | |
| A0A0B5EMG9 | Efflux pump FUBT | Membrane efflux pump for fusaric acid export. Virulence factor. | AlphaFold | NA | 83.94% | Autosite | |
| P23618 | Thiamine thiazole synthase | Thiamine precursor biosynthesis. Converts NAD and glycine to ADT. | AlphaFold | NA | 92.94% | Residues: 175, 230, 103, 223, 296, 226, 110 (AlphaFill) | |

pLDDT: predicted Local Distance Difference Test score for AlphaFold models (range 0-100%). Scores >80% indicate reliable structural predictions. NA: Not apply.
