# Supplementary material for: Antifungal activity of Burkholderia sensu lato metabolites against coffee phytopathogens: evidence of chemical diversity
Source: World J Microbiol Biotechnol. 2026 Sep 27;42(10):556. doi: 10.1007/s11274-026-05282-5 (PMC13616859; doi:10.1007/s11274-026-05282-5)
Supplement: Supplementary file 7 [file 11274_2026_5282_MOESM9_ESM.docx]

**Table S3.** Molecular docking scores of bioactive compounds against selected fungal target proteins.

| Compound | A0A0B5EMG9 | C7C435 | P9WER6 | P23295 | P23618 | P35049 | P39863 | P46237 | P78717 | Q8X1D8 | Q9C1K0 | Q96UG7 |
| --- | --- | --- | --- | --- | --- | --- | --- | --- | --- | --- | --- | --- |
| Occidiofungin (C_54_H_78_N_12_O_19_) | -7.8 ± 0.0 | **-8.1** | 71.6 | -7.6 ± 1.1 | **-8.8** ± 0.7 | -7.1 ± 0.3 | 128.6 | -7.4 ± 0.0 | -4.8 | 1.6 | 172.6 | **-8.2** ± 0.4 |
| Burkholdine (C_52_H_83_N_11_O_22_) | -5.8 ± 0.0 | -3.3 ± 0.0 | 91.1 | -1.9 | -6.1 ± 0.0 | -5.5 ± 0.0 | 112.7 | -6.8 | -3.8 | -0.9 ± 2.9 | 174.6 | -4.4 |
| Burkholdine (C_52_N_11_O_22_H_85_) | -5.8 | -4.8 | 92.4 | 2.9 | -4.8 ± 0.7 | -6.6 | 91.6 | **-8.7** | -2.7 | 3.4 | 200.1 | -5.5 |
| Icosalide  (C_34_H_60_N_4_O_10_) | -6.0 | -6.0 ± 0.3 | -2.0 | **-8.1** ± 0.5 | -5.6 ± 0.6 | -5.7 ± 0.5 | -1.6 ± 1.3 | -7.0 ± 1.0 | -5.8 ± 0.0 | -5.7 ± 0.7 | 53.8 | -5.3 ± 0.6 |
| Icosalide  (C_36_H_64_N_4_O_10_) | -7.0 ± 0.3 | -5.5 ± 0.6 | -4.1 | -7.6 ± 0.7 | -6.9 ± 0.3 | -5.8 ± 0.5 | 9.6 | -6.9 ± 0.2 | -6.2 ± 0.7 | -4.6 ± 0.0 | 48.1 | -6.2 ± 0.7 |
| Lagriamide (C_41_H_68_N_2_O_10_) | **-10.0** | -7.9 | -7.1 | -7.6 | **-10.6** | **-9.0** | **-10.1** ± 3.9 | **-9.8** | -7.0 | **-9.3** ± 1.1 | 23.5 | **-10.2** |
| Burkholdine (C_52_H_83_N_11_O_23_) | -4.7 | -5.5 | 69.3 | -1.3 ± 0.0 | -4.7 ± 0.0 | -3.5 | 98.3 | -5.5 | -2.3 | 12.1 | 191.0 | -4.3 ± 1.6 |
| Burkholdine (C_47_H_75_N_11_O_19_) | -7.0 ± 1.0 | -4.7 | 39.4 | -4.1 ± 1.8 | -4.0 ± 0.6 | -4.7 ± 0.0 | 76.2 | -7.2 ± 1.2 | -5.2 | -2.3 ± 4.3 | 161.8 | -3.1 ± 0.8 |
| Burkholdine (C_52_H_85_N_11_O_22_) | -5.5 | -3.6 | 102.0 | -7.9 | -3.9 ± 0.0 | -4.6 | 110.0 | -3.2 | -1.9 | -0.6 ± 1.2 | 191.7 | -3.8 ± 0.0 |
| Occidiofungin (C_52_N_11_O_21_H_84_Cl) | -6.4 | -7.1 | 112.0 | -1.2 ± 1.1 | -5.3 | -5.7 | 111.0 | -6.0 | -2.4 | 4.3 | 188.9 | -4.7 ± 0.0 |

Values are expressed as mean ± SD, where multiple docking runs were performed. **Bold green** values indicate strong binding (≤ −8.0 kcal/mol); red values indicate failed/unfavorable docking (positive ΔG); — = not calculated*.* Protein identifiers correspond to UniProt accession numbers.
